# Simultaneous quantification of nine aconitum alkaloids in *Aconiti Lateralis Radix Praeparata* and related products using UHPLC–QQQ–MS/MS

**DOI:** 10.1038/s41598-017-13499-6

**Published:** 2017-10-12

**Authors:** Fan He, Can-Jian Wang, Ying Xie, Chun-Song Cheng, Zhong-Qiu Liu, Liang Liu, Hua Zhou

**Affiliations:** 1 State Key Laboratory of Quality Research in Chinese Medicine, Macau University of Science and Technology, Macau, China; 2 Faculty of Chinese Medicine, Macau University of Science and Technology, Macau, China; 3 Department of Chinese Medicine Analysis, Liaoning University of Traditional Chinese Medicine, Dalian, China; 4 International Institute of Translation Chinese Medicine, Guangzhou University of Chinese Medicine, Guangzhou, China

## Abstract

*Aconiti Lateralis Radix Praeparata* (Fuzi) is obtained from processed daughter roots of Aconitum carmichaeli, a toxic plant with a high medical value well known in Chinese medicine. In addition to the known toxic alkaloids (aconitine, mesaconitine, and hypaconitine) and bioactive alkaloids (benzoylaconine, benzoylmesaconine, and benzoylhypaconine), three rarely found alkaloids have been previously reported in Fuzi, i.e., yunaconitine, 8-deacetyl-yunaconitine, and crassicauline A, and they were reported in recent years to cause potential risk to patients who took Fuzi or related products. To better control the quality of this herb and its related products and ensure safe use, developing a method to simultaneously determine these 9 alkaloids is important. In this research, sensitive and accurate ultra-high-performance liquid chromatography coupled with triple quadrupole mass spectrometry method was established and used to examine 51 Fuzi and 27 Fuzi-containing products. Unexpectedly, 8-deacetyl-yunaconitine was detected in 17 Fuzi samples (33.3%) and 3 Fuzi-containing products (11.1%); yunaconitine in 10 Fuzi samples (19.6%) and 10 Fuzi-containing products (37.0%); and crassicauline A in 3 Fuzi samples (5.8%). Industry and clinics should be aware of the unusually high detection rate of these three toxic alkaloids in the Fuzi herb and its related products and take the necessary precautions to protect patients from any potential risk.

## Introduction

*Aconiti Lateralis Radix Praeparata* (Fuzi in Pinyin), the processed daughter root of *Aconitum carmichaeli* Debx. (Family *Ranunculaceae*), is a well-known Chinese medical material recorded in the Chinese Pharmacopoeia (CHP)^1^ and other traditional Chinese medicine (TCM) texts. Fuzi has been used for over 2000 years as an analgesic and anti-inflammatory agent in clinics^2^. According to TCM theories, Fuzi is very toxic^2^. Modern experimental studies have shown that diester-diterpenoid alkaloids (DDAs) are the active toxic ingredients in Fuzi. These toxic DDAs have cardiac, analgesic, and anti-inflammatory activities^3–5^. To reduce the toxicity, crude Fuzi must be processed by heating or steaming, and processed Fuzi must be boiled before oral administration according to TCM theories and practice. During these processes, aconitine (AC), mesaconitine (MA), and hypaconitine (HA), which are the main toxic DDAs in Fuzi, degrade into less toxic but still active monoester-diterpenoid alkaloids (MDAs), such as benzoylaconine (BAC), benzoylmesaconine (BMA), and benzoylhypaconine (BHA), respectively^6^. Therefore, 6 aconitum alkaloids have been selected in the CHP as the target elements for the quality control of aconitum herbs. According to the CHP (2015 Edition), the total amount of AC, MA and HA in Fuzi must be less than 0.020%, and the total amount of BAC, BMA and BHA must be higher than 0.010%. Nevertheless, poisoning cases are still occasionally reported. From 1989 to 2010, 140 cases of aconitum poisoning, including one fatal case, were reported in Hong Kong^7^. Additionally, 17 cases were reported in Taiwan from 1990–1999, 2017 cases in China from 1989–2008, and 121 cases in Korea from 1995–2007^8^. Multiple reasons for aconitum poisoning exist and include over doses, inadequate processing, aconitum contamination in other herbs, dispensing and management errors, and hidden risk factors^7^. In the 17 cases reported in Hong Kong, yunaconitine (YAC), crassicauline A (CCA), and 8-deacetyl-yunaconitine (DYA) were detected instead of AC, MA and HA in the urine samples of the aconitum poisoning patients^7,9^. Although YAC and CCA are two DDAs with toxicities similar to AC^10^, they are mainly isolated from *A. forrestii*, *A. crassicaule*, and other aconitum species^9^, not from *A. carmichaeli*. Therefore, the surveillance of toxic alkaloids from aconitum herbs always neglects YAC and CCA. DYA is the hydrolytic product of yunaconitine. Although DYA is less toxic than YAC, it still presents a safety risk because of the conversion between the two compounds^11^. Because YAC, DYA and CCA were detected in the urine of the aconitum poisoning patients, these alkaloids are considered to be hidden risk factors and should be covered in laboratory screenings for toxic compounds^9^. Therefore, a method to simultaneously determine the levels of these 9 alkaloids is needed for quality control of the herb and its products and to ensure the safe use of these medical materials.

Although the high-performance liquid chromatography (HPLC) and liquid chromatography hyphenated with mass spectrometry (LC/MS) methods have been broadly applied for the quantification of aconitum alkaloids in herb or herbal products and body fluids over the past few years^12–18^, the maximum number of aconitum alkaloids simultaneously determined using these methods is only 7, i.e., AC, MA, HA, BAC, BMA, BHA, and YAC. The challenge in simultaneously determining the 9 aconitum alkaloids is that the level of some these alkaloids are significantly lower than the detection limits of the current methods. In the present study, ultra-high-performance liquid chromatography coupled with triple quadrupole mass spectrometry (UHPLC–QQQ–MS/MS) was used to simultaneously determine the contents of 9 aconitum alkaloids in Fuzi and Fuzi-containing products. The method was rapid, sensitive, accurate and fully validated and was used to analyze 51 Fuzi samples and 27 Fuzi-containing product samples Table 1. In addition to quality control for Fuzi and its preparations, this method is also valuable for toxicological and forensic studies of related samples.

## Results

### UHPLC-MS optimization

Different solvents and gradient profiles of the mobile phase were compared to achieve a good resolution within 15 min. Acetonitrile and an acidic aqueous solution significantly improved the resolution and the symmetry of the target compounds. Acetonitrile-0.1% formic acid was used as the mobile phase with the gradient mentioned in the Materials and methods section at a flow rate of 0.35 mL/min.

**Table 1:** Sample information for the Fuzi samples and the related products.

Sample number	Sample name	Source (region)
FZ01-FZ13	Aconiti Lateralis Radix Praeparata	Hong Kong
FZ14-FZ15	Aconiti Lateralis Radix Praeparata	Jiangxi Province
FZ16-FZ20	Aconiti Lateralis Radix Praeparata	Hong Kong
FZ21-FZ23	Aconiti Lateralis Radix Praeparata	Changsha, Hunan Province
FZ24-FZ26	Aconiti Lateralis Radix Praeparata	Gansu Province
FZ27-FZ28	Aconiti Lateralis Radix Praeparata	Bozhou, Anhui Province
FZ29-FZ30	Aconiti Lateralis Radix Praeparata	Zhuhai, Guangdong Province
FZ31-FZ32	Aconiti Lateralis Radix Praeparata	Guangzhou, Guangdong Province
FZ33-FZ35	Aconiti Lateralis Radix Praeparata	Yunnan Province
FZ36-FZ38	Aconiti Lateralis Radix Praeparata	Dalian, Liaoning Province
FZ39-FZ40	Aconiti Lateralis Radix Praeparata	Macau
FZ41-FZ43	Aconiti Lateralis Radix Praeparata	Hong Kong
FZ44-FZ45	Aconiti Lateralis Radix Praeparata	Butuo, Sichuan Province
FZ46	Aconiti Lateralis Radix	Butuo, Sichuan Province
FZ47	Aconiti Lateralis Radix	Butuo, Sichuan Province
FZ48-FZ50	Aconiti Lateralis Radix Praeparata	Taiwan
FZ51	Aconiti Lateralis Radix Praeparata	Hong Kong
HSP01-HSP11	Heishunpian granule	Hong Kong
DFP01-DFP03	Danfupian granule	Shenzhen
BFZ	Baifuzi granule	Shenzhen
PFP	Paofupian granule	Shenzhen
YGW01-YGW02	You-gui-wan	Shenzhen
SNT	Si-ni-tang	Shenzhen
MFXT01-MFXT02	Ma-huang-fu-zi-xi-xin-tang	Shenzhen
ZWT	Zhen-wu-tang	Shenzhen
FLT	Fu-zi-li-zhong-tang	Shenzhen
JGSQ01-JGSQ02	Jin-gui-shen-qi-wan	Shenzhen
XSLJT01-XSLJT02	Xiang-sha-liu-jun-zi-tang	Shenzhen

**Figure 1:**
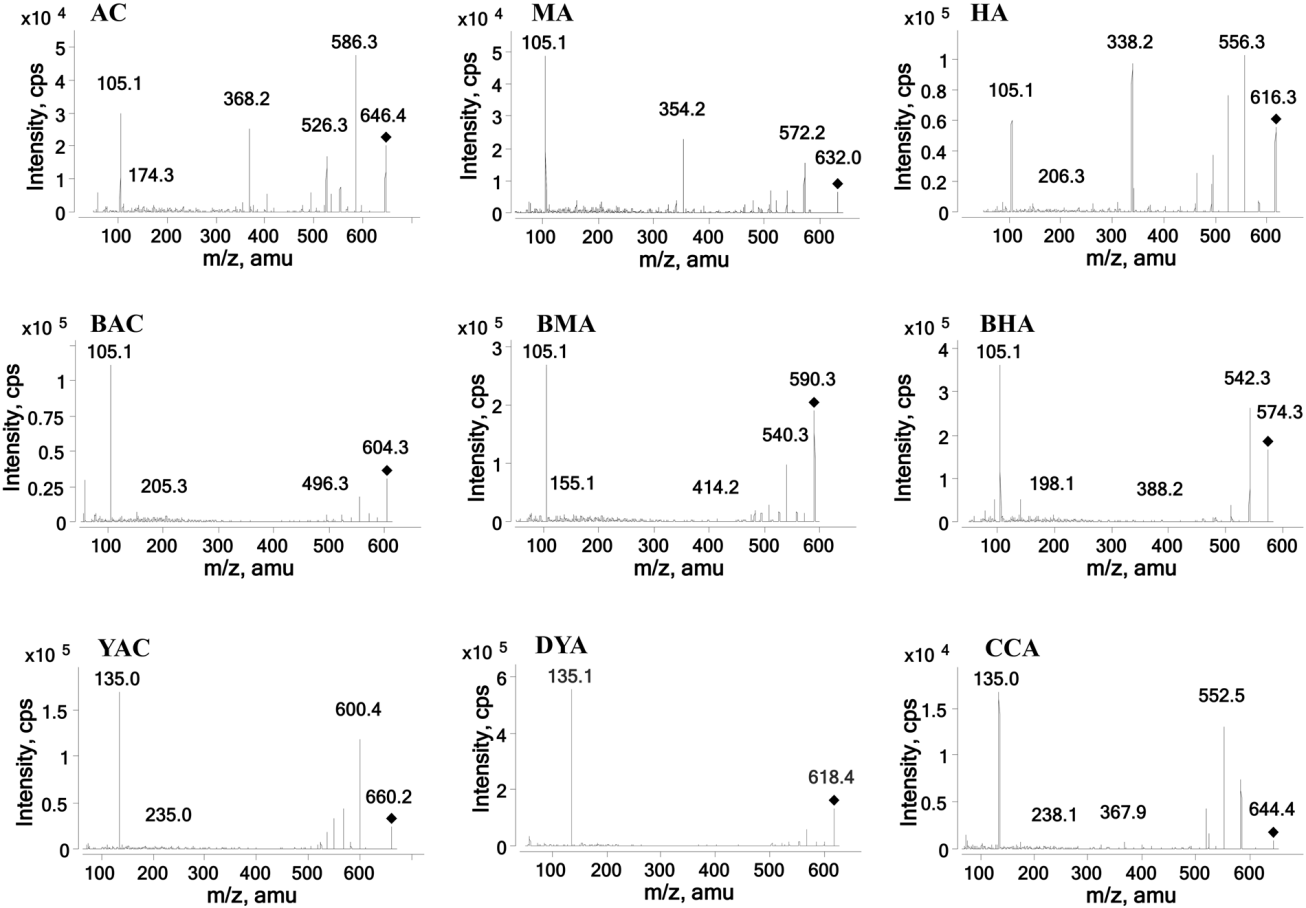
Full scan product ion mass spectra of the 9 compounds.

The negative and positive ion modes were compared for the MS analysis. The positive mode resulted in a higher sensitivity and cleaner mass spectral background than the negative mode. The collision energy and fragmentor voltage parameters were optimized to obtain the highest relative abundance of the exclusive ions and production in the multiple reaction monitoring (MRM) optimization conditions. The final conditions for the collision energy and fragmentor voltage are shown in the Instruments and UHPLC-MS conditions section. The MS/MS ion spectra of the 9 alkaloids are shown in Fig. 1.

### Method validation

**Figure 2:**
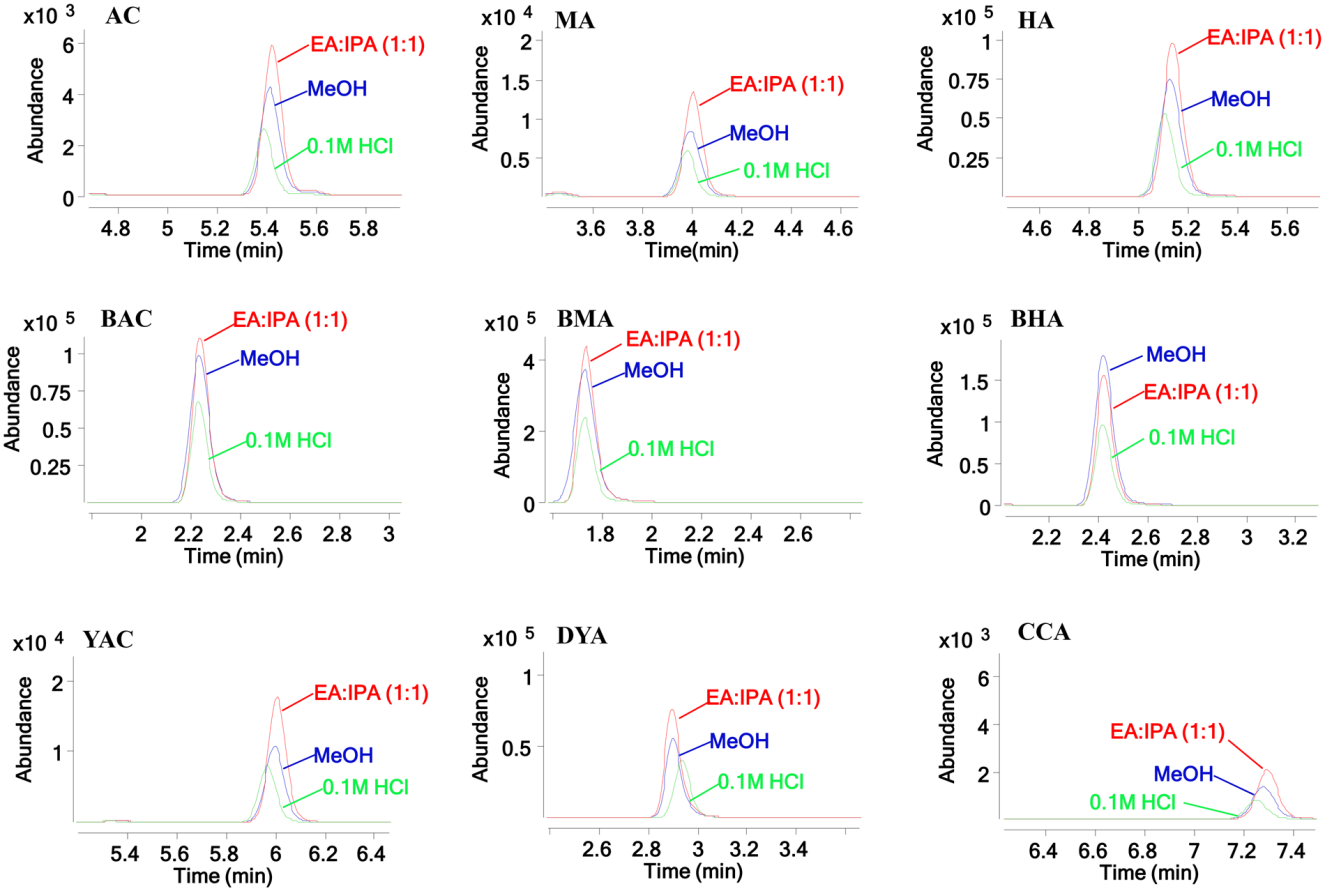
Extraction efficiency of the 9 compounds in the Fuzi samples using different solvents.

The extraction method, including the extraction solvent and extraction times, was optimized to effectively extract the 9 alkaloids with a large difference in the content levels for the UHPLC-MS analysis. A 0.1 M hydrochloric acid solution (50 mL), 50 mL of methanol with 3 mL of the ammonia test solution (ammonia TS, prepared by adding water to 400 mL of a 28% ammonia solution to make 1000 mL, according to the CHP), and 50 mL of isopropanol (IPA) and ethyl acetate (EA) (1:1) with 3 mL of the ammonia TS were compared as the extraction solvents. Ethyl acetate and isopropanol (1:1) with 3 mL of ammonia TS were the most efficient for extracting the alkaloids (Figure 2 and Table 2). The extraction times were also compared. One, two, and three times for the repeated extraction resulted in insignificant differences. Therefore, a single extraction was selected for this analysis. The optimal sample preparation method was extracting 2 g of sample with 50 mL ethyl acetate and isopropanol (1:1) and 3 mL ammonia TS in an ultrasonicator for 30 min.

**Table 2:** The peak areas of the 9 aconitum alkaloids in the Fuzi sample (FZ06) using the extraction method.

Extraction methods	AC	MA	HA	BAC	BMA	BHA	YAC	DYA	CCA
EA:IPA (1:1)	30906	68567	552857	524633	2171283	750078	92461	357995	12834
MeOH	23947	50014	460300	504798	2070036	988550	60098	255022	8767
0.1 M HCl	13272	28606	284949	311592	1116647	465404	41436	191209	4919

The calibration curves with at least 5 different concentrations were analyzed. Table 3 lists the linear calibration curves with the R value, linear range, LOQ, LOD, and repeatability. Table 4 lists the precision, stability and recovery of the 9 alkaloids. A signal-to-noise ratio (S/N) of approximately 3 was set as the LOD, and a S/N of 10 was set as the LQD. All the calibration curves showed good linear ranges (R > 0.999) within the respective ranges and were adequate for the determinations of the 9 alkaloids in the samples.

**Table 3:** Linearity, range and limits of determination and quantification.

Analytes	Linearity	R	Range (ng/mL)	LOD (ng/mL)	LOQ (ng/mL)
AC	Y = 20447X + 6476	0.9997	0.656–6560	0.131	0.656
MA	Y = 19008X + 4958	0.9997	0.546–5460	0.109	0.546
HA	Y = 18548X + 8882	0.9995	0.789–7890	0.158	0.789
BAC	Y = 22899X + 9210	0.9994	0.565–5650	0.113	0.565
BMA	Y = 19125X + 31020	0.9990	1.65–16500	0.330	1.650
BHA	Y = 24881X + 10680	0.9993	0.591–5910	0.118	0.591
YAC	Y = 385584X + 5933	0.9998	0.495–4950	0.099	0.495
DYA	Y = 42795X + 8856	0.9998	0.508–5080	0.102	0.508
CCA	Y = 45812X − 1659	1.0000	0.253–2530	0.051	0.253

**Table 4:** Precision, repeatability, stability and recovery of the 9 compounds.

Analyte	Precision RSD (%) (n = 6)	Repeatability RSD (%) (n = 6)	Stability RSD (%) (n = 6)	Recovery				
				Original (μg)	Spiked (μg)	Found (μg)	Recovery (%)	RSD (%)
AC	2.90	1.52	1.95	1.72	1.73	3.42	98.2	2.54
MA	1.80	1.75	2.11	3.42	3.56	7.04	101.7	2.49
HA	2.35	1.79	1.55	50.8	50.3	100	98.9	3.30
BAC	1.39	1.36	1.54	52.4	52.3	104	99.1	2.65
BMA	0.818	1.38	1.04	222	220	442	100.1	2.35
BHA	2.55	1.47	0.369	83.2	83.1	165	99.2	1.71
YAC	1.32	1.05	1.38	1.08	1.12	2.19	98.8	3.32
DYA	1.29	1.36	1.77	5.66	5.66	11.3	100.1	2.73
CCA	1.36	1.86	1.67	0.584	0.592	1.17	99.5	2.92

The precision was determined by measuring one concentration level six times in the same day. The repeatability was tested using six samples that were prepared using the same sample and method. The stability was determined over periods of 0, 2, 4, 6, 8 and 12 h in one day. The results (Table 4) indicated that the method for the investigated samples had good precision and reproducibility. In addition, the compounds were sufficiently stable and could be routinely analyzed within 12 h at room temperature.

The recovery was examined by spiking a certain amount (1 g) of a Fuzi sample with a known amount of the mixed standards (n = 6). The recoveries were calculated using the equation below:

Recovery (%) = (total amount detected−amount original)/amount spiked × 100%

As shown in Table 4, the recoveries of the 9 alkaloids ranged from 98.2% to 101.7%, and the RSD values were less than 4.0%, which showed the high quantification accuracy.

### Sample determination

**Figure 3:**
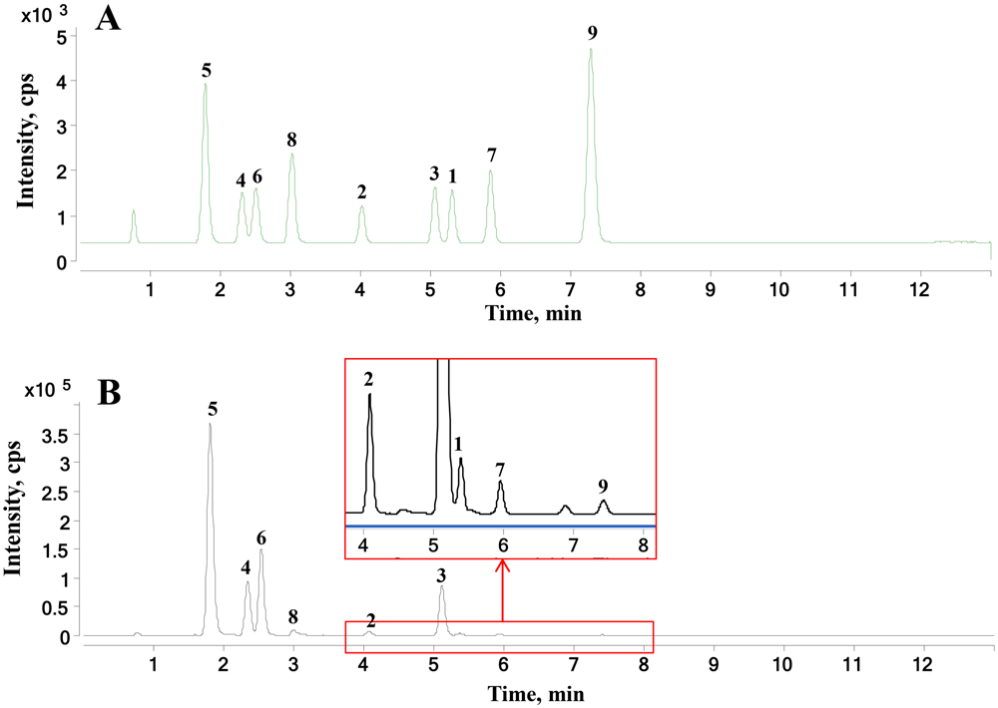
Representative MRM chromatograms of the reference standard mixture (**A**) and Fuzi samples (**B**). 1, AC; 2, MA; 3, HA; 4, BAC; 5, BMA; 6, BHA; 7, YAC; 8, DYA; 9, CCA.

**Figure 4:**
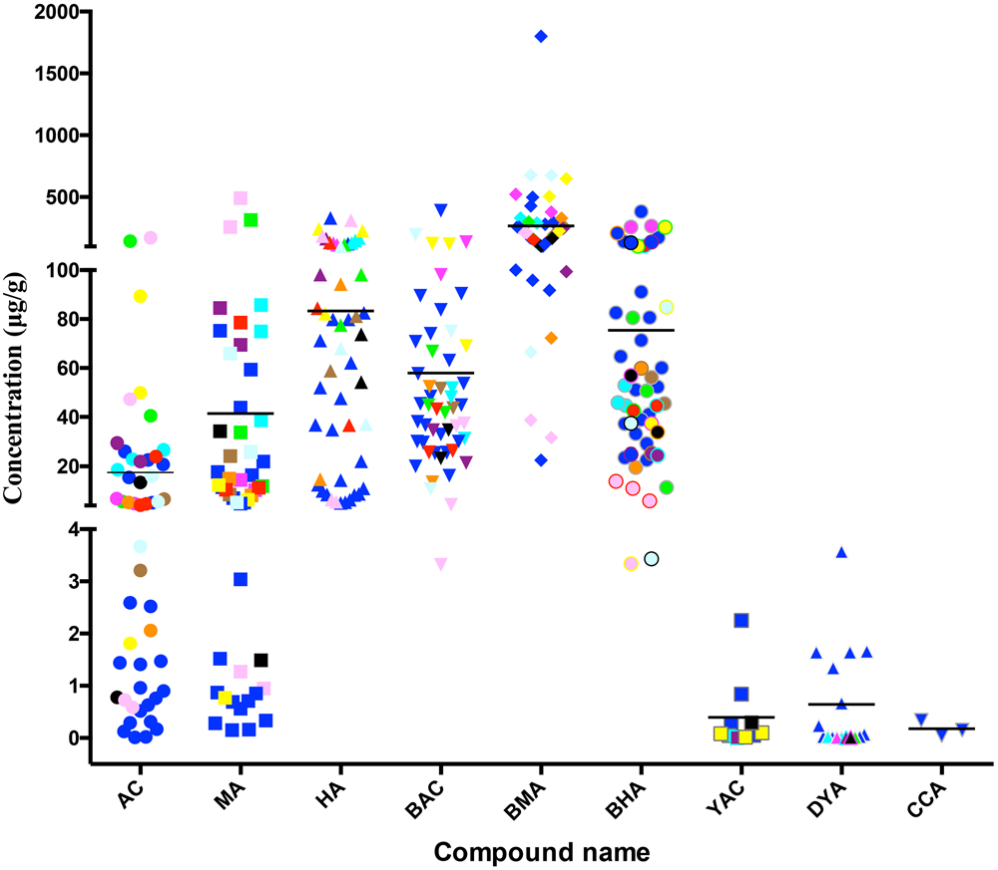
Distribution of the content of the 9 compounds in the Fuzi samples from different regions of P.R. China. Blue: Hong Kong, brown: Jiangxi, cyan: Changsha, green: Gansu, magenta: Bozhou, orange: Zhuhai, purple: Guangzhou, red: Yunnan, yellow: Dalian, black: Macao, pale magenta: Butuo, pale cyan: Taiwan. The black line represents the mean concentration of each compound.

The established method was successfully used for the simultaneous determination of 9 alkaloids in 51 Fuzi samples and 27 Fuzi-containing products. The representative MRM chromatograms of the reference standard mixture and the Fuzi sample are shown in Fig. 3. The contents of the 9 alkaloids are summarized in Table 5, and the content distribution of the 9 alkaloids in different regions of China is shown in Fig. 4.

**Table 5:** The contents of the 9 aconitum alkaloids in the Fuzi samples and Fuzi-containing products.

Sample number	AC (μg/g)	MA (μg/g)	HA (μg/g)	BAC (μg/g)	BMA (μg/g)	BHA (μg/g)	YAC (μg/g)	DYA (μg/g)	CCA (μg/g)	Total contents (%) AC + MA + HA	Total contents (%) BAC + BMA + BHA
FZ01	2.52	11.5	62.1	53.7	296	80.6	—	1.66	—	0.00761	0.0430
FZ02	1.41	7.03	52.0	48.4	259	71.3	—	1.64	—	0.00604	0.0379
FZ03	0.170	0.710	8.53	83.8	22.5	41.0	—	—	—	0.000941	0.0147
FZ04	15.5	59.3	79.9	30.0	199	23.6	—	0.0106	—	0.0155	0.0253
FZ05	0.0200	0.690	14.4	25.3	95.9	52.3	—	—	—	0.00151	0.0174
FZ06	0.630	5.25	71.2	57.7	244	82.6	0.838	3.57	0.0454	0.00771	0.0384
FZ07	5.16	17.7	82.6	63.1	279	91.1	0.0530	1.64	—	0.0105	0.0433
FZ08	20.8	75.2	9.98	36.7	211	29.1	—	0.0677	—	0.0106	0.0277
FZ09	0.963	22.0	79.9	38.1	115	64.7	—	—	—	0.0103	0.0218
FZ10	0.0100	0.160	47.6	44.8	196	60.1	0.277	1.34	0.152	0.00478	0.0301
FZ11	26.0	16.4	123	45.4	233	37.2	—	0.0256	—	0.0165	0.0316
FZ12	22.6	43.8	330	390	1800	382	—	0.233	—	0.0396	0.257
FZ13	1.44	3.04	22.1	32.9	162	33.1	—	—	—	0.00266	0.0228
FZ14	3.21	8.50	58.9	51.6	196	56.2	—	—	—	0.00706	0.0304
FZ15	6.64	24.2	81.2	43.6	258	45.5	—	—	—	0.0112	0.0347
FZ16	0.127	0.153	5.04	16.2	91.8	22.6	—	—	—	0.000532	0.0131
FZ17	0.519	0.870	12.5	25.5	171	38.9	—	—	—	0.00139	0.0235
FZ18	0.314	0.335	11.0	29.6	103	51.1	—	—	—	0.00116	0.0184
FZ19	0.899	1.52	8.32	70.8	242	172	—	—	—	0.00107	0.0485
FZ20	1.47	0.28	5.31	90.4	499	139	—	0.0262	—	0.000706	0.0728
FZ21	18.6	38.6	169	31.2	125	44.7	0.0455	0.0191	—	0.0226	0.0201
FZ22	26.6	85.7	129	48.1	291	45.9	—	0.00483	—	0.0241	0.0385
FZ23	22.9	75.0	149	51.8	331	52.9	—	—	—	0.0247	0.0436
FZ24	40.5	33.7	77.6	44.8	146	50.7	—	0.0105	—	0.0152	0.0242
FZ25	5.59	11.9	114	41.8	158	80.5	—	—	—	0.0131	0.0280
FZ26	144	314	98.2	66.8	299	11.4	—	—	—	0.0556	0.0377
FZ27	4.76	14.5	127	98.1	379	257	—	—	—	0.0146	0.0734
FZ28	6.79	10.3	113	136	522	265	—	0.00708	—	0.0130	0.0923
FZ29	2.06	8.13	14.7	13.5	72.3	19.6	—	—	—	0.00249	0.0105
FZ30	5.24	15.0	94.3	52.4	329	59.9	—	—	—	0.0115	0.0441
FZ31	29.4	69.5	164	21.4	99.4	25.19	—	—	—	0.0263	0.0146
FZ32	22.0	84.5	98.2	34.6	247	24.45	0.00711	0.00648	—	0.0205	0.0306
FZ33	24.0	78.6	133	25.6	164	104	—	—	—	0.0236	0.0294
FZ34	4.13	10.5	36.7	43.2	133	44.6	—	—	—	0.00513	0.0221
FZ35	4.68	11.3	84.4	26.3	141	42.6	—	—	—	0.0100	0.0210
FZ36	1.81	0.770	82.2	119	649	255	0.0151	—	—	0.00848	0.102
FZ37	49.9	6.34	243	123	505	102	0.0988	—	—	0.0299	0.0730
FZ38	89.4	12.3	227	69.0	226	37.3	0.0824	—	—	0.0329	0.0332
FZ39	0.779	1.49	54.2	23.3	103	56.9	0.290	0.0102	—	0.00565	0.0183
FZ40	13.4	34.2	73.7	34.6	165	33.8	—	—	—	0.0121	0.0233
FZ41	2.59	4.79	34.8	74.0	256	208	—	—	—	0.00422	0.0538
FZ42	0.290	0.560	8.78	20.0	100	24.9	—	—	—	0.000963	0.0145
FZ43	—	—	6.39	30.0	119	135	—	—	—	0.000639	0.0284
FZ44	0.585	0.949	6.47	37.4	212	13.9	—	—	—	0.000800	0.0263
FZ45	0.726	1.27	5.53	36.3	213	11.0	—	—	—	0.000753	0.0260
FZ46	170	490	312	4.33	31.6	5.89	—	—	—	0.0972	0.00418
FZ47	47.3	258	184	3.32	38.7	3.34	—	—	—	0.0489	0.00454
FZ48	3.67	5.28	68.0	194	679	84.9	—	—	—	0.00770	0.0958
FZ49	16.0	65.9	37.0	10.7	66.5	3.43	—	—	—	0.0119	0.00807
FZ50	5.50	25.8	101	75.0	674	37.4	—	—	—	0.0133	0.0786
FZ51	0.762	0.851	36.8	89.5	428	131	2.25	0.665	0.337	0.0038	0.0649
**Minimum**	0.0100	0.153	5.04	3.32	22.5	3.34	0.00711	0.00483	0.0454		
**Maximum**	170	490	330	390	1800	382	0.838	3.57	0.152		
**Average**	17.8	42.2	84.2	57.3	263	74.3	0.190	0.642	0.099		
**Median**	4.76	11.5	75.65	43.4	205	48.3	0.0824	0.0259	0.0987		
HSP01	—	—	0.566	45.2	259	110	3.00	—	—		
HSP02	—	—	1.05	63.7	336	147	4.26	—	—		
HSP03	0.794	2.65	31.7	24.0	152	40.1	—	—	—		
HSP04	—	—	0.067	43.4	241	103	—	—	—		
HSP05	0.0481	0.140	6.11	14.6	70.3	46.8	—	—	—		
HSP06	0.266	—	—	37.7	312	121	—	—	—		
HSP07	—	0.00660	0.272	54.7	333	92.8	0.141	—	—		
HSP08	—	0.375	11.2	74.3	389	199	1.34	—	—		
HSP09	0.130	1.21	59.2	77.3	299	112	—	—	—		
HSP10	—	0.191	6.95	108	197	144	1.64	—	—		
HSP11	—	—	0.551	37.6	280	35.7	—	—	—		
DFP01	—	—	0.655	33.3	211	63.2	—	—	—		
DFP02	—	0.482	—	0.081	—	0.591	—	—	—		
DFP03	0.368	0.289	—	0.0876	0.919	1.27	—	—	—		
BFZ	—	0.0658	0.164	0.256	2.17	0.404	—	—	—		
PFP	—	—	0.340	10.7	37	17.7	—	—	—		
YGW01	—	—	0.122	0.758	5.8	1.47	0.0302	—	—		
YGW02	0.0258	1.97	0.400	2.19	13.6	4.15	0.388	0.0328	—		
SNT	—	—	1.04	7.04	45.1	13.8	0.0981	—	—		
MFXT01	—	0.0145	1.79	14.6	83.9	27.8	0.276	0.0912	—		
MFXT02	—	0.0214	2.35	11.6	70.1	21.9	0.402	0.0886	—		
ZWT	—	0.0798	1.09	5.69	31.1	14.5	—	—	—		
FLT	—	0.0811	2.35	11.6	70.1	5.72	—	—	—		
JGSQ01	—	—	0.0324	0.677	0.317	1.31	—	—	—		
JGSQ02	0.374	1.10	1.75	1.60	9.69	1.97	—	—	—		
XSLJT01	—	—	—	—	—	—	—	—	—		
XSLJT02	—	—	—	—	—	—	—	—	—		
**Minimum**	0.0258	0.00660	0.0324	0.0810	0.317	0.404	0.0302	0.0328			
**Maximum**	0.794	2.65	59.2	108	389	199	4.26	0.0912			
**Average**	0.287	0.578	5.90	27.2	144	53.1	1.16	0.0709			
**Median**	0.266	0.191	1.045	14.6	77.1	27.8	0.395	0.0886			

Notes:

“—” under the lower limit of detection.

FZ, *Aconiti Lateralis Radix* Praeparata; HPS: Heishunpian granule; DFP: Danfupian; BFZ: Baifuzi; YGW: You-gui-wan; SNT: Si-ni-tang; MFXT: Ma-huang-fu-zi-xi-xin-tang; ZWT: Zhen-wu-tang; FLT: Fu-zi-li-zhong-wan; JGSQ: Jing-gui-shen-qi-wan.

AC: aconitine; MA: mesaconitine; HA: hypaconitine; BAC: benzoylaconine; BMA: benzoylmesaconine; BHA: benzoylhypaconine; YAC: yunaconitine; CCA: crassicauline A; DYA: 8-deacetyl-yunaconitine.

## Discussion

An optimized method for quantifying the main constituents in pharmaceutical preparations is important for quality control standards to ensure the safety, quality and effectiveness of Chinese herbal products. In this study, 3 active alkaloids and 6 toxic alkaloids in Fuzi were selected as chemical markers for quality control. A total of 51 samples, 49 processed Fuzi and 2 unprocessed Fuzi samples, were collected from 10 areas in China, including 8 provinces and 2 special administrative regions, namely, Guangdong (Zhuhai, Guangzhou), Hunan (Changsha), Anhui (Bozhou), Jiangxi, Gansu, Liaoning (Dalian), Yunnan, and Taiwan Provinces, as well as Hong Kong and Macao, providing a good geographical distribution of the samples.

**Figure 5:**
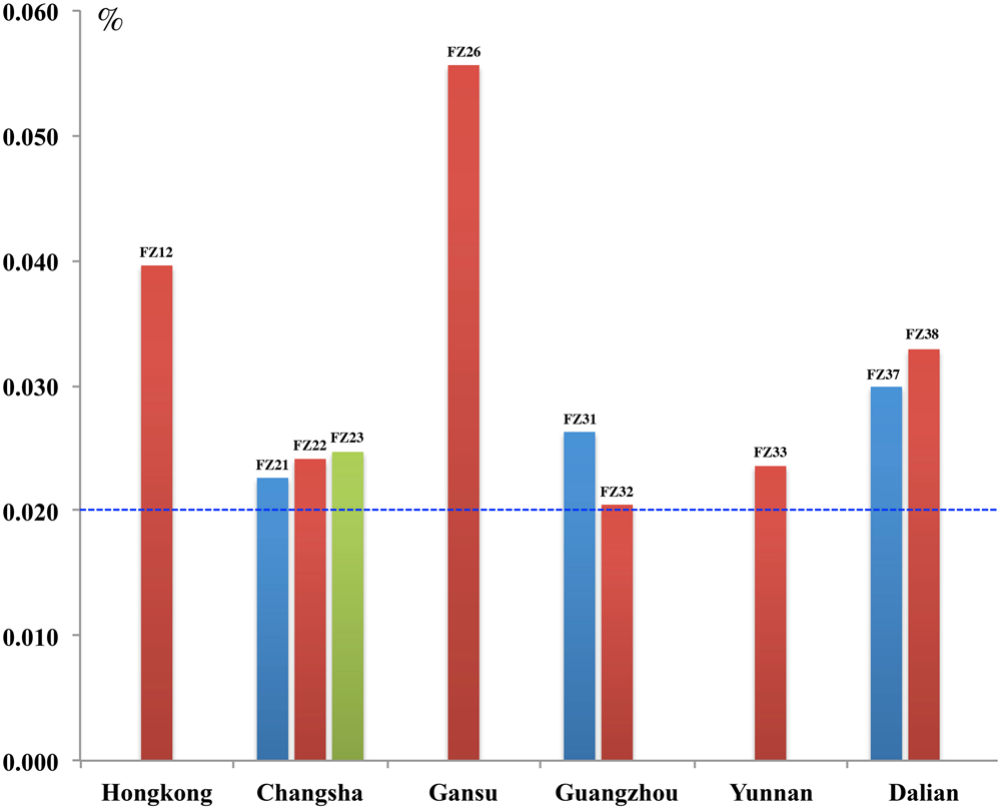
The AC, MA and HA contents in 10 batches from five places that were beyond the limits set by the Chinese Pharmacopoeia. Blue dotted line: the upper limit of the total amounts of AC, MA and HA according to the Chinese Pharmacopoeia, i.e., less than 0.020%.

The total concentrations of BAC, BMA, and BHA in the 49 processed Fuzi samples complied with the CHP criteria except for one sample (no. FZ49) that was below the CHP limits. In these 49 samples, the contents of AC, MA and HA were beyond the CHP limits in 10 batches; i.e., approximately 20.4% of the processed Fuzi samples did not meet the requirements of the national pharmacopoeia of China, which implies a quality control matter for processed Fuzi. The contents of the six aconitum alkaloids (AC, MA, HA, BAC, BMA and BHA) in the two unprocessed aconitum roots (No. FZ46 and FZ47) were above the CHP limits, showing that aconitum roots need to be processed to ensure safety. The unqualified Fuzi samples were collected from Hong Kong (one batch, No. F12), Changsha (3 batches, No. F21, F22 and F23), Gansu Province (one batch, No. F26), Guangzhou (2 batches, No. F31 and F32), Yunnan Province (one batch, No. F33) and Dalian (2 batches, No. F37 and F38) (Fig. 5).

**Figure 6:**
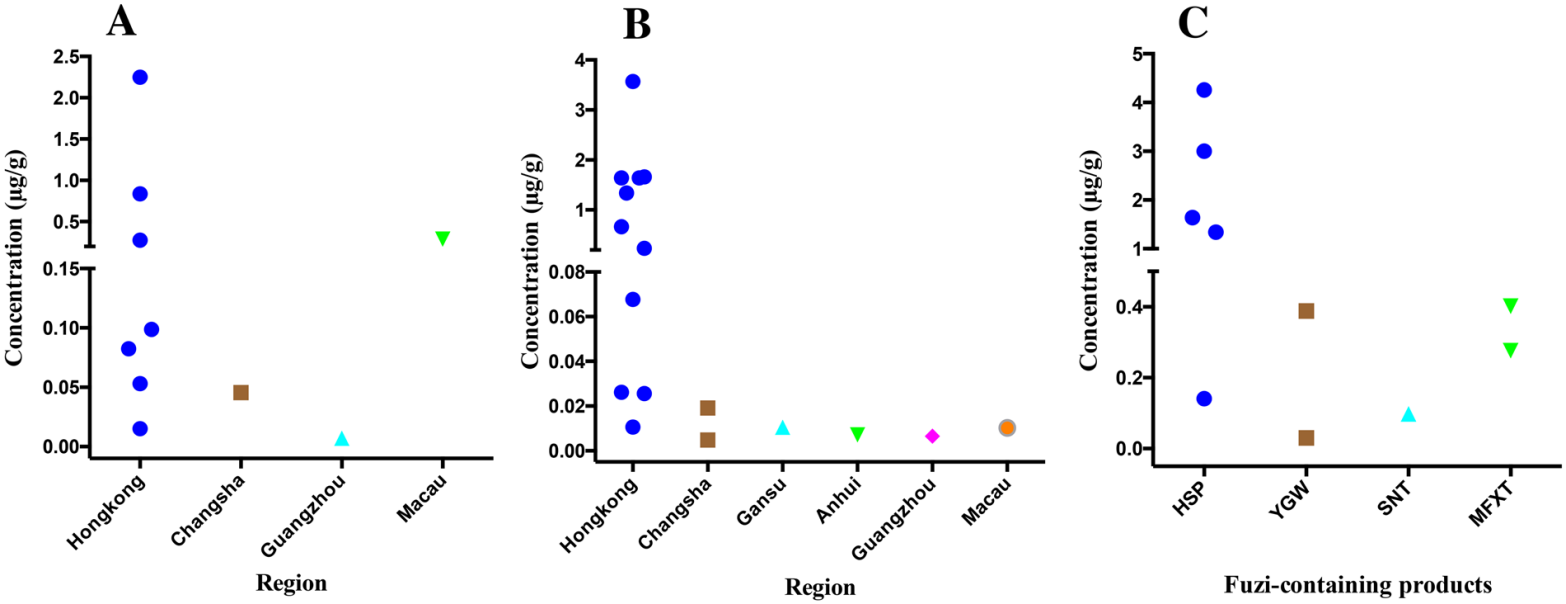
The contents of the three toxic alkaloids (YAC, DYA and CCA) in some of the Fuzi samples and Fuzi-containing products. (**A**) The YAC content in the Fuzi samples and the distribution area, (**B**) the DYA content in the Fuzi samples and the distribution area, (**C**) the YAC content in Fuzi-containing products from Hong Kong.

YAC was detected in 10 of the 51 Fuzi samples at concentrations ranging from 0.00711 to 2.25 μg/g. These samples were collected from five places, including Hong Kong, Changsha, Guangzhou, Dalian and Macao. Meanwhile, DYA was detected in 17 Fuzi samples at concentrations ranging from 0.00483 to 3.57 μg/g. Figures 6A and B show the contents of YAC and DYA in Fuzi samples from different areas, and the contents were highest in the samples from Hong Kong. CCA was detected in 3 samples (No. FZ06, FZ10 and FZ51 from Hong Kong) at concentrations of 0.0454, 0.152 and 0.337 μg/g, respectively. Three Fuzi samples (approximately 5.88%, no. FZ06, FZ10 and FZ51) had all three alkaloids (YAC, DYA and CCA). Four samples (approximately 7.84%, No. FZ07, FZ21, FZ32 and FZ39) contained trace amounts of both YAC and DYA.

**Figure 7:**
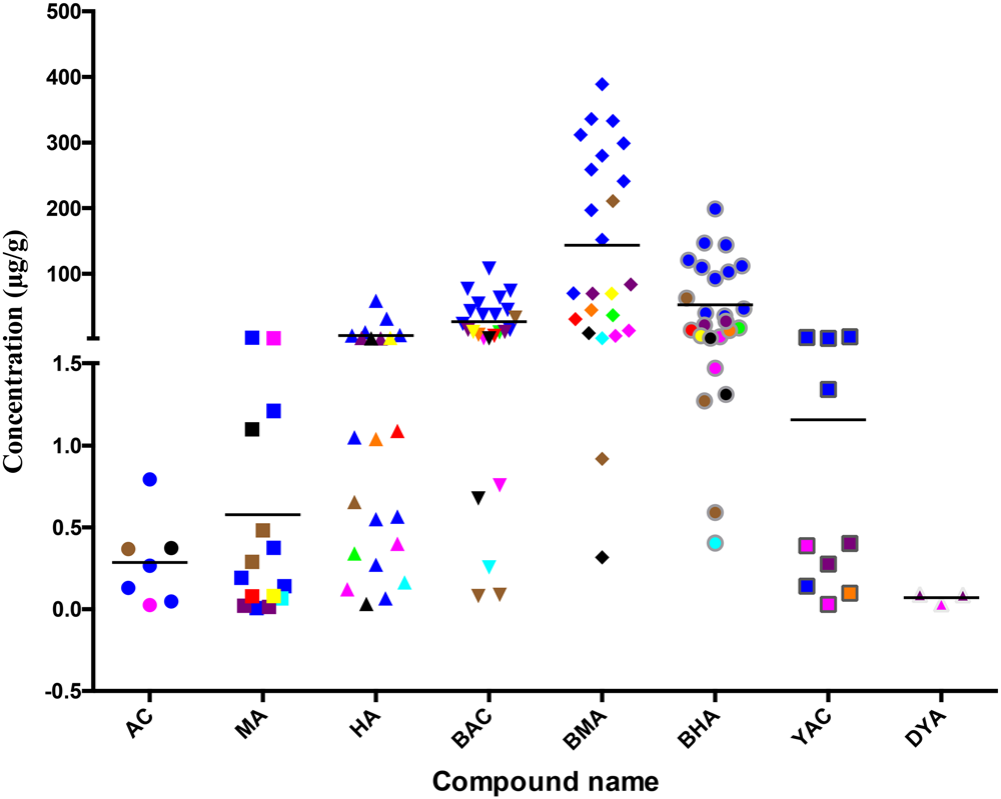
Distribution of the content of the 9 compounds in the Fuzi-containing products from Hong Kong. Blue: HPS, brown: DFP, cyan: BFZ, green: PFP, magenta: YGW, orange: SNT, purple: MFT, red: ZWT, yellow: FLT, black: JGSQ. The black line represents the mean concentration of each compound.

In addition, 27 samples of Fuzi-containing products were also analyzed. YAC was found in 10 samples (Fig. 6C), and a trace amount of DYA was detected in 3 samples (No. YGW02, MFXT01 and MFXT02). In addition, the distributions of the contents of the 9 components in all the Fuzi-containing products are shown in Fig. 7.

YAC, DYA and CCA were detected in one third of the Fuzi samples (17/51) and 37% of the Fuzi-containing products (10/27). DYA was the most frequently detected alkaloid in the Fuzi samples, and YAC was the most frequently detected alkaloid in the Fuzi-containing products. Both compounds have a high detection rate, which has not been reported before. YAC, DYA and CCA are toxic aconitum alkaloids, and the acute toxicity of YAC is comparable to that of aconitine^18^. The detection of the three alkaloids at such a high rate implies that there may be unknown risks for patients who consume Fuzi or Fuzi-containing products. One of the major alkaloids in *A. vilmorinianum*, *A. hemsleyanum* and *A. nagarum* is YAC^19^. The roots of the above species are occasionally misrepresented as *Radix Aconiti Kusnezoffii* and used as “Caowu” (in Chinese) in Chinese herbal medicine. In addition, the detection of YAC was reported in some herbs that are closely related to Fuzi, such as *Aconiti Radix Preparata*, *Aconiti Kusnezoffii Radix*, and *Aconiti Kusnezoffii Radix Preparata*, and products containing these herbs, but not in Fuzi^14^. The previously published literature also implies that the determination of YAC, DYA and CCA contents in Fuzi and Fuzi-containing products is a safety issue when Fuzi and its products are used in clinics.

In recent years, the frequency of aconitum poisoning has increased, and this could be a result of the YAC, DYA and CCA contents in processed Fuzi, as discovered by the current study. Currently, the YAC or CCA contents in aconitum are unknown, and only two studies have reported the isolation of YAC and DYA from Fuzi^20,21^. However, the Fuzi source was not clearly presented in the reports, and the evidence was insufficient to draw a conclusion. Therefore, a possible reason for the detection of YAC, DYA and CCA in Fuzi could be contamination. YAC and CCA, which are mainly found in certain aconitum roots from Southwest China, were most commonly detected^22^. Fuzi may be contaminated by aconitum roots via accidental contamination, misidentification or even intentional adulteration. In the production, storage, packaging and transport of herbal medicines, unwanted introduction of foreign substances, including toxic plants or weeds, can occur (World Health Organization, 2007). Determining how non-toxic herbs are contaminated with aconitum roots is not easy. Thus, strengthening the source control through good agricultural and supply practices and appropriate quality assurance is important.

In this study, a method for determining 9 aconitum alkaloids in Fuzi samples was established and validated, providing an important reference for the quality control of Fuzi and research on related toxic components, such as YAC. Does Fuzi contain YAC and other ingredients, and what content levels should be set for safe use? These questions should be further investigated in future pharmacological and toxicological studies. The existing information on the quality control of YAC, DYA and CCA in Fuzi and Fuzi-containing products is insufficient. In this work, these 3 alkaloids were all detected in commercial Fuzi samples and Fuzi-containing products, implying that YAC, DYA and CCA might be hidden toxic ingredients and should be used as quality control and toxicological monitoring indicators. In addition, limit levels for the three alkaloids should be set after careful and thorough studies on their safety and pharmacological effects. The current findings should indicate to manufacturers of Fuzi and Fuzi-containing products that strictly controlling the quality of these medicinal materials is important.

## Materials and Methods

### Chemicals, reagents and materials

Fuzi samples were purchased from local drug stores or herb markets in different places in P.R. China, including Guangdong, Liaoning, Hunan, Yunan, Jianxi, Anhui, Sichuan Provinces and Hong Kong, Macao, and Taiwan. All these materials were authenticated by Dr. Zhi-Feng Zhang (An expert of herbal authentication at Macau University of Science and Technology). Voucher specimens were deposited in the State Key Laboratory of Quality Research in Chinese Medicine (Macau University of Science and Technology). The Fuzi-containing products used in this study were Heishunpian granule (HSP), Danfupian granule (DFP), Baifuzi granule (BFZ), Paofupian granule (PFP), You-gui-wan (YGW), Si-ni-tang (SNT), Ma-huang-fu-zi-xi-xin-tang (MFXT), Zhen-wu-tang (ZWT), Fu-zi-li-zhong-tang (FLT), and Jin-gui-shen-qi-wan (JGSQ) and were purchased from local pharmacies in Shenzhen and Hong Kong P.R. China. Xiang-sha-liu-jun-zi-tang (XSLJZT) that is free of Fuzi was used as a negative control. The sample information, including the sample number, sample name and sample source, is shown in Table 1.

**Figure 8:**
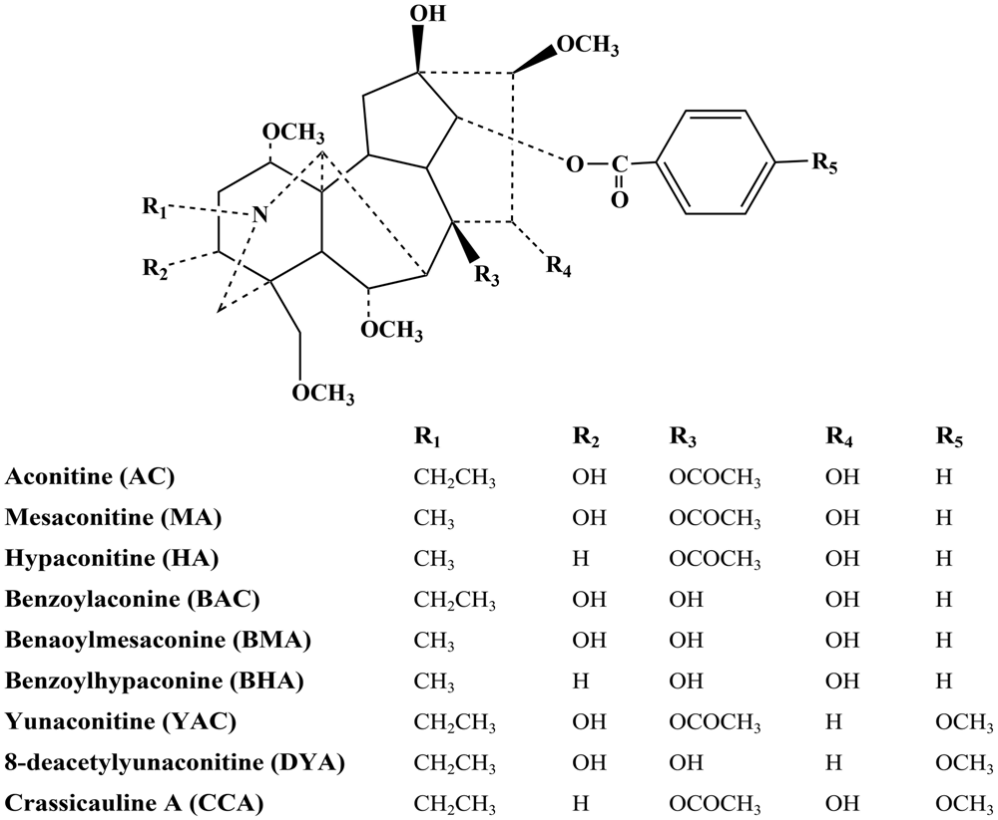
Chemical structures of the 9 standard reference compounds.

The reference standard compounds (purity ≥ 98%), aconitine, hypaconitine, mesaconitine, benzoylaconine, benzoylhypaconine, benzoylmesaconine, and crassicauline A, were purchased from the Chengdu Must Bio-technology Co., Ltd. (Chengdu, P.R. China). Yunaconitine (purity ≥ 98%) was purchased from the Testing Laboratory for Chinese Medicine (Hong Kong, P.R. China). 8-Deacetyl-yunaconitine (purity ≥ 98%) was kindly provided by Prof. Xiao-Xia Ma of Yunnan University of TCM (P.R. China). The structures are given in Fig. 8. Formic acid (St. Louis, MO, USA), acetonitrile and methanol (Houston, TX, USA) were all HPLC grade. Other chemicals and reagents were of analytical grade.

### Instruments and UHPLC-MS conditions

An UHPLC system (1290 series, Agilent Technologies, Santa Clara, CA, USA) coupled with an Agilent 6460 Triple Quadrupole Mass Spectrometer (QQQ-MS, Agilent Technologies, Santa Clara, CA, USA) was used to quantitatively detect the 9 aconitum alkaloids. Chromatographic separations were performed on a Waters Acquity UPLC C_18_ column (1.7 μm, 2.1 mm × 100 mm, Waters, Milford, MA, USA) at 30 °C. The mobile phase consisted of 0.1% acetic acid (A) and acetonitrile (B), and the gradient elution was as follows: 20–25% B from 0-4 min, 25% B from 4–10 min, 90% B from 10.01–12 min, and 20% B from 12.01–15 min. An aliquot of 2 μL was injected, and the flow rate was 350 μL/min. The detection of the 9 alkaloids was performed using MRM and an ESI source in a positive ion mode. The transitions of the 9 compounds were m/z 646.4 → 586.3 (Frag 184, CE33) for AC, m/z 632.0 → 105.1 (Frag 184, CE 50) for MA, m/z 616.3 → 556.3 (Frag 184, CE 29) for HA, m/z 604.3 → 105.1 (Frag 242, CE 50) for BAC, m/z 590.3 → 105.1 (Frag 184, CE 49) for BMA, m/z 574.3 → 105.1 (Frag 184, CE 50) for BHA, m/z 660.2 → 135.0 (Frag 205, CE 64) for YCA, m/z 618.4 → 135.1 (Frag 235, CE 52) for DYA, and m/z 644.4 → 135.0 (Frag 220, CE 68) for CCA. The other parameters were as follows: drying gas (N_2_) flow rate, 11.0 L/min; drying gas temperature, 300 °C; nebulizer, 15 psig; and capillary voltage, 4000 V.

### Standard solution preparation

**Figure 9:**
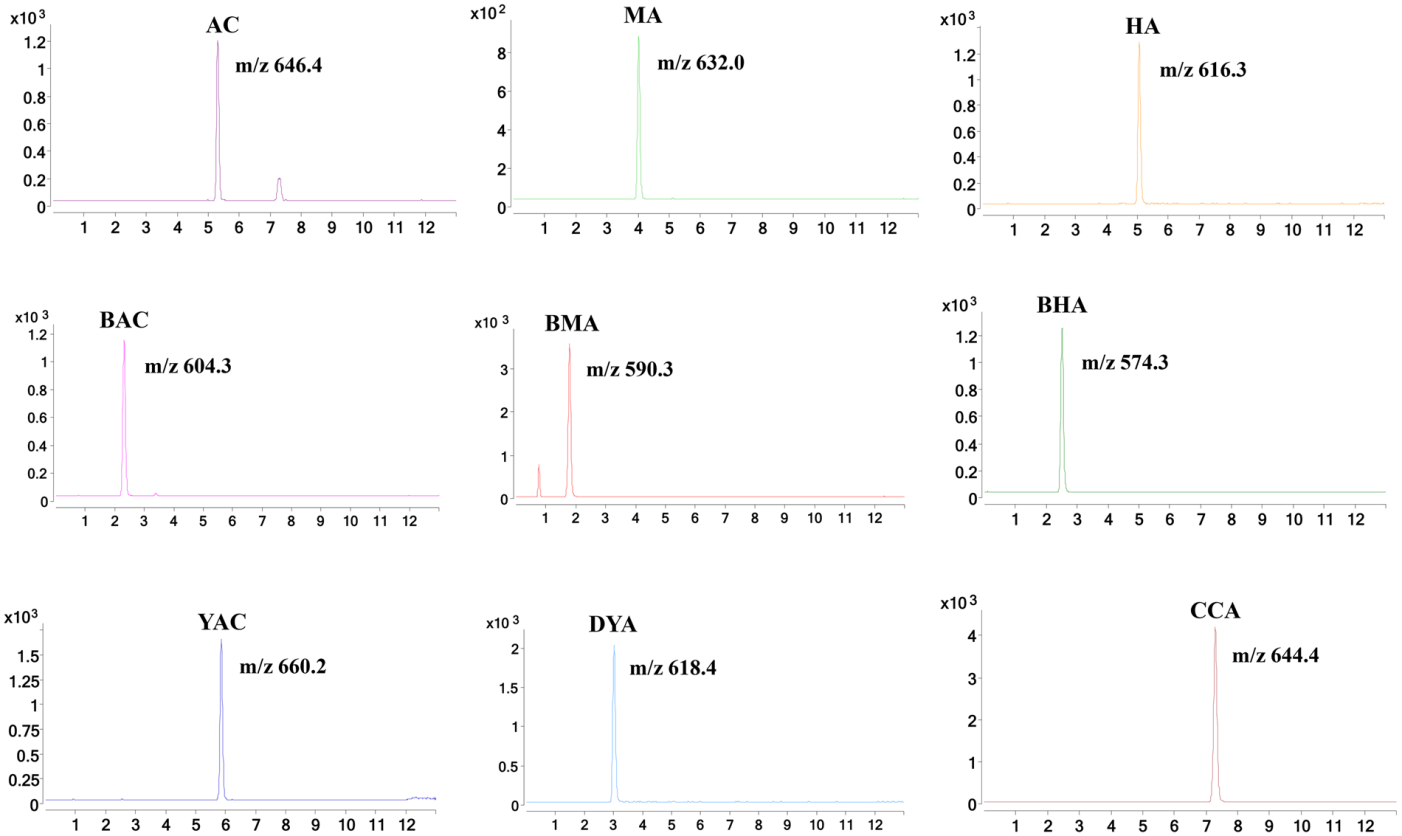
UHPLC–QQQ–MS/MS EIC of the 9 reference standard compounds.

An appropriate amount of the 9 reference standards was dissolved with methanol to prepare stock solutions, which were stored at 4 °C. The standard mixture solution was obtained by accurately mixing the 9 stock solutions and diluting them with 50% methanol. The final concentrations of AC, MA, HA, BAC, BMA, BHA, YAC, DYA, and CCA in the mixture solution were 6.56, 5.46, 7.89, 5.65, 16.5, 5.91, 4.95, 5.08 and 2.53 μg/mL, respectively. The different concentrations of the standard solutions in Table 3 were obtained by diluting the mixture solution. Two microliters of the standard solution were injected into the UHPLC-MS system, and the total ion chromatogram charts of the 9 reference standards are shown in Fig. 9.

### Method validation and preparation of the sample solutions

The method was validated in accordance with the Guidelines for the Validation of Quality Standard of TCM (CHP, 2015 Edition, Volume 1)^1^ and the US Food and Drug Administration bioanalytical method validation (US Food and Drug Administration, 2001)^23,24^.

The Fuzi powder (2.0 g) was accurately weighed and ultrasonically extracted for 30 min using 3 mL of ammonia TS and 50 mL of ethyl acetate-isopropanol (1:1, v/v) in a conical flask that was covered after the solution cooled to room temperature. The loss in weight was supplemented via the addition of more extraction solvent. The extraction was filtered, and 25 mL of the successive filtrate was evaporated to dryness below 40 °C. The residue was precisely dissolved in 3 mL of isopropanol- dichloromethane (1:1, v/v). The solution was then diluted with methanol in a 1:10 ratio and filtered with a 0.22 μm microporous membrane filter. Two microliters of the successive filtrate was injected into the UHPLC-MS system for the analysis, according to the method mentioned in the Instruments and UHPLC-MS conditions section.
